# Practical and psychosocial challenges faced by caregivers influence the acceptability of multidrug-resistant tuberculosis preventive therapy for young children

**DOI:** 10.1371/journal.pone.0268560

**Published:** 2022-07-14

**Authors:** Dillon T. Wademan, Graeme Hoddinott, Susan E. Purchase, James A. Seddon, Anneke C. Hesseling, Anthony J. Garcia-Prats, Ria Reis, Lindsey J. Reynolds

**Affiliations:** 1 Faculty of Medicine and Health Sciences, Department of Paediatrics and Child Health, Desmond Tutu TB Centre, Stellenbosch University, Stellenbosch, South Africa; 2 Department of Infectious Diseases, Imperial College London, London, United Kingdom; 3 Department of Paediatrics, University of Wisconsin, School of Medicine and Public Health, Madison, Wisconsin, United States of America; 4 Department of Public Health & Primary Care, Leiden University Medical Centre, Leiden, Netherlands; 5 Amsterdam Institute for Social Sciences, University of Amsterdam, Amsterdam, The Netherlands; 6 The Children’s Institute, University of Cape Town, Cape Town, South Africa; 7 Faculty of Arts and Social Sciences, Department of Sociology and Social Anthropology, Stellenbosch University, Stellenbosch, South Africa; 8 Pivot Collective, Cape Town, South Africa; ICMR-National Institute for Research in Tuberculosis: National Institute of Research in Tuberculosis, INDIA

## Abstract

Drug-resistant (DR) strains of *Mycobacterium tuberculosis* (*M*. *tb*) are increasingly recognised as a threat to global tuberculosis (TB) control efforts. Identifying people with DR-TB exposure/ infection and providing TB preventive therapy (TPT) is a public health priority. TB guidelines advise the evaluation of household contacts of newly diagnosed TB cases, with the provision of TPT to vulnerable populations, including young children (<5 years). Many children become infected with TB through exposure in their household. Levofloxacin is under evaluation as TPT in children exposed to *M*. *tb* strains with resistance to rifampicin and isoniazid (multidrug-resistant TB; MDR-TB). Prior to opening a phase 3 prevention trial in children <5 years exposed to MDR-TB, the pharmacokinetics and safety of a novel formulation of levofloxacin given daily was evaluated as part of a lead-in study. We conducted an exploratory qualitative study of 10 caregivers’ experiences of administering this formulation. We explored how the acceptability of levofloxacin as TPT is shaped by the broader impacts of MDR-TB on the overall psychological, social, and financial wellbeing of caregivers, many of whom also had experienced MDR-TB. Caregivers reported that the novel levofloxacin formulation was acceptable. However, caregivers described significant psychosocial challenges in the process of incorporating TPT administration to their children into their daily lives, including financial instability, withdrawal of social support and stigma. When caregivers themselves were sick, these challenges became even more acute. Although new child-friendly formulations can ameliorate some of the pragmatic challenges related to TPT preparation and administration, the overall psychosocial burden on caregivers responsible for administering TPT remains a major determinant of effective MDR-TB prevention in children.

## Introduction

In 2019, approximately 10 million people developed tuberculosis (TB) disease and 1.4 million people died of TB, making it the leading infectious cause of death worldwide [1]. The World Health Organization (WHO) estimated that close to half a million people developed rifampicin-resistant (RR) TB, of whom 78% had multidrug-resistant (MDR)-TB (defined as disease caused by strains of *Mycobacterium tuberculosis* (*M*. *tuberculosis*) with resistance to at least isoniazid and rifampicin). MDR-TB, even when diagnosed and treated, is associated with poor treatment outcomes in adults [1]. Approximately 30,000 children (<15 years) develop MDR-TB disease each year and, although treatment outcomes are better for children when promptly diagnosed, treatment can be long and challenging [2].

Following exposure to drug-susceptible (DS) TB, TB preventive therapy (TPT) is universally recommended for all people with substantial risk of progression to disease, including for children <5 years [1,3]. Multiple TPT regimens are available for children exposed to DS-TB and recently child-friendly regimens and formulations have been developed [4]. In contrast, the evidence for TPT following MDR-TB exposure with proven efficacy is limited, and there is limited guidance from the WHO regarding preventive chemotherapy for children exposed to MDR-TB [5,6].

Most young children are infected with *M*. *tuberculosis* in their household, following exposure through parents or other older household members [7]. Household contact tracing activities involve screening household members for TB disease with the provision of TPT to those without disease but at high risk of future disease progression [4,8]. Children’s caregivers play an essential role in uptake, administration and adherence to all child-targeted interventions, including TPT [9,10]. Recent research suggests that caregivers’ perceptions of TPT influence their willingness to procure and administer TPT to their children [11–13]. If caregivers delay treatment administration or are unable to comply with treatment guidelines, children are at increased risk of progression to TB disease and potentially death [14]. Treatment acceptability, defined as “the overall ability of the patient and caregiver (defined as ‘user’) to use a medicinal product as intended (or authorised)”, [15] has become a central concern to childhood antituberculosis treatment [16,17]. However, evidence suggests there are many other determinants of TPT uptake in young children exposed to MDR-TB [13,18].

Caregivers with MDR-TB in particular experience multiple social, economic and psychological barriers to their own treatment adherence including stigma, health systems barriers, catastrophic costs related to care, and psychological distress [19–24]. These same challenges can also compromise their ability to effectively care for children [25]. This situation is further complicated when a child is at risk of acquiring TB from their caregiver, potentially leading to feelings of guilt and experiences of blame [25,26]. The withholding of social support by members of their social support network can further impair their capacity for care [26,27]. However, the ways in which these complex factors affect the overall acceptability of administering MDR TPT to children has not been explored to date.

TB-CHAMP [28] is a clinical trial to evaluate whether levofloxacin can prevent TB disease in child (<5 years) household contacts of individuals with MDR-TB. Prior to trial initiation, a novel, dispersible, child-friendly formulation of levofloxacin was developed by Macleods Pharmaceuticals (Mumbai, India). During this pre-trial phase, a questionnaire-based study was conducted to evaluate the palatability of this novel formulation [17]. Concurrently, we conducted a qualitative sub-study to contextualise and triangulate the survey results, by exploring acceptability more in-depth—including palatability, ease of administration, and social determinants of care. Specifically, we describe the palatability of the formulation and caregivers’ experiences of treatment administration, and then contextualise these relative to the caregivers’ own psychosocial circumstances and experiences of TB disease.

## Methods

### Setting

TB-CHAMP is a phase III cluster randomised placebo-controlled trial to assess the efficacy of levofloxacin in young child contacts (<5 years of age) of MDR-TB implemented at three sites in South Africa [28]. Before the trial began, the study team conducted a pharmacokinetic lead-in study to determine appropriate dosages and preliminary palatability and acceptability of the novel levofloxacin formulation in 27 children [17,28,29]. Although an internationally accepted standard approach to preventive therapy for children exposed to MDR-TB does not exist, a regimen consisting of levofloxacin (adult 250 mg formulation), ethambutol and high-dose isoniazid is prescribed to children exposed to MDR-TB in Cape Town [17]. Children recruited were household contacts <5 years of age of MDR-TB index cases who temporarily interrupted the standard of care regimen. Children were then started on weight-banded doses (15–20 mg/kg) of the novel, dispersible formulation of levofloxacin, once-daily for 7–14 days before resuming routine treatment for the remainder of their 6 months TPT. Caregivers administered the treatment to their children. The study-specific levofloxacin formulation could be administered whole, crushed, or dissolved in water and given with or without food.

We report here on qualitive data regarding children and caregivers’ experiences of the novel, dispersible levofloxacin formulation collected between January and March 2017 in Cape Town, South Africa. The Western Cape had the highest overall incidence of TB of all provinces in 2015, estimated at 681 new cases per 100 000 [30]. A prospective surveillance study of confirmed MDR-TB in children attending a tertiary hospital in Cape Town, between 2013–2017, reported an estimated prevalence of between 7.1% and 8.9% [2].

### Sampling and recruitment

We employed an ethnographic method to understand what meaning caregivers conferred upon levofloxacin during treatment administration and other related care interactions with their children and the health system. The ethnographic method is well-suited to this endeavour as it encourages researchers to see participants’ engagement with the phenomenon being studied (in this case levofloxacin) as ‘symbolic action,’ and to attempt to understand the meanings attributed to the phenomenon [31]. Researchers and participants are together involved in a process of ‘meaning making,’ as the phenomenon under study is constructed and reconstructed at every interaction. This approach is, therefore, inherently subjective in terms of the knowledge it produces, because there is a distinction between the phenomenon and the terms and theories researchers use to describe and understand them [32].

The unit of recruitment was the child and their caregiver(s). We sampled purposively for diversity in child age and gender and continued until theoretical saturation in the participants’ experiences of the formulation ingestion and administration. We also conducted 27 days of semi-structured observations in the unit where study staff administered the levofloxacin formulation to children and demonstrated administration to the caregivers. During this observation period, we approached potential participants who were enrolled in the lead-in study and who had been suggested as potential participants by the clinical trial team. We interacted with participants at each of their study visits over the 7–14-day period on study, resulting in each child-caregiver dyad unit being observed three or four times (responsive to other demands on their time).

Thereafter a graduate qualitative researcher contacted the participant and arranged a time and place suitable to the participant (usually their home or a separate room in the trial site) to conduct an interview. Although the child-caregiver dyad were the primary participants, caregivers’ partners and other household members were sometimes present during the interviews. These other household members would typically continue with their daily tasks and drift in and out of the conversation for short periods. However, the caregivers remained the primary participants and respondents during interviews. In two cases the interviews were conducted after participants’ close-out visit of the lead-in study and after restart of the standard of care treatment regimen. In total, we interviewed 4 boys and 8 girls with two sets of siblings, totalling 10 child-caregiver dyads. Six of the caregivers had MDR-TB at the time of data collection.

### Data collection

Data were collected by two socio-behavioural science graduate researchers. The first author (DW) was always present and was supported by a fellow researcher fluent in the participant’s preferred language. Researchers collecting data were experienced research assistants holding an honours or masters-level degree in social or behavioural science. The interviews ranged from 15 to 90 minutes, were conducted in the participants’ preferred languages (English, Afrikaans, or Xhosa), and used a semi-structured discussion guide. Each interviewer had extensive experience of using interviews towards data collection and underwent study-specific training to ensure high-quality data were captured. Additionally, each interviewer had prior experience working with caregivers of children on treatment. Interviewers endeavoured to supplant any potential social barriers with caregivers through multiple, extended interactions before scheduling the interview. The analysis was led by DW. It was important to recognise that his interpretative lens was shaped by being a white South African male. This will have impacted on the understanding of the complexities of administering TPT to children exposed to TB (usually by household members, including the interviewee). Participants’ responses may have been constrained by desirability bias or perceived authority over healthcare access. Further, being unable to speak all participants’ home languages may have limited the ability of the lead analyst to understand cultural nuance and thus impacted the quality of data collected. Conversely, his relative ignorance may have allowed probing/clarification questions which may have otherwise been overlooked. Additionally, being accompanied by an experienced researcher speaking the participants’ home language may have further negated potential social barriers.

Topics in the interview discussion guides included participants socio-demographic details including their community and household structures, the caregivers’ knowledge and perceptions of TB disease and experiences of administering levofloxacin. All interviews were recorded using a digital recording device. The interview recordings were then transferred to a central, secure computer located at Desmond Tutu TB Centre (DTTC) for analysis preparation. All interviews underwent a five-step transcription process, comprising (1) initial verbatim transcription (2) checking for transcription accuracy, (3) initial translation to English, (4) checking for translation accuracy, and (5) anonymisation. While conducting the household interviews, researchers also completed a structured observation tool [33].

### Data analysis

The data were analysed through an iterative, inductive thematic analysis process [34]. This approach aims to identify, capture, and describe the central themes and common experiences shared by participants. DW and LR led the analysis process, with support from GH and RR. From an ethnographic perspective, theory does not accurately represent ‘reality’ but provides a useful lens through which to interpret participant’s meaning making. Therefore, the analysis was not guided by a pre- defined theoretical framework *per se*, as it was *inductive* (not deductive) and thus shaped by what the data revealed. Rather, the ‘acceptability’ of paediatric treatments (see references [16,35–37]) were conceptually engaged to guide interpretation. To do so, all the interview transcripts were read and re-read to identify common experiences influencing treatment acceptability across the broader dataset. Exemplary cases, which served to highlight these experiences were read in greater detail alongside the notes and observational data for those cases. Shared experiences across the data set were then amassed into the themes presented in the findings section. Although strict triangulation of the data was not possible, triangulation through the data collection and analysis process, was operationalised in two ways: (a) field notes of both interviewers present at data collection were written separately and then reviewed together to clarify perspectives and, (b) interpretation of interviews was achieved through the iterative analysis process where multiple analysis (co-authors) interpreted the data and discussed these interpretations.

### Ethics

The study was approved by the Health Research Ethics Committee at Stellenbosch University (M16/02/009), national authorities and local health authorities. All caregiver participants completed written informed consent prior to participation. Caregivers’ informed consent included consent for the child in their care to be included in observations and interview discussions. Confidentiality was retained through de-identifying participants from the data at the point of data capturing, as well as assigning participants pseudonyms at the point of data transcription.

## Findings

Overall, the levofloxacin in children had relatively high acceptability. Caregivers in our study reported that they were willing to administer this formulation to their well children and showed a clear understanding of TPT. Children and caregivers also said that they preferred the levofloxacin formulation over other TB treatment drugs and formulations that they had used or seen their family members use in the past–describing this formulation as more palatable. However, even when using this novel levofloxacin formulation, caregivers experienced pragmatic difficulties integrating TPT for their children into their lives. These difficulties revolved primarily around the financial and care burden due to TPT experienced by the household. These challenges were exacerbated for caregivers who were concurrently on treatment for their own MDR-TB disease, limiting their capacity to care for their children. Caregivers with MDR-TB described how stigma, shame, and the withdrawal of social support by family and friends further hindered their ability to ensure that their children received TPT as prescribed.

### Willingness to initiate and adhere to TPT

All the caregivers interviewed in this study said they were willing to administer levofloxacin to their well children to prevent them from developing MDR-TB in future. In their explanations of their motivations for administering TPT to their children, caregivers showed a good understanding of TB transmission and risk and of the potential benefits of TPT. One caregiver, whose landlord was being treated for MDR-TB, explained her motivation to initiate her child on TPT:

I stay with this person [being treated for MDR-TB], and I am with him most of the time, so the child must be protected. [The nurses] said it’s for her protection. They didn’t find TB in the child. […] I understand it from the point of view that she must be protected, so she doesn’t get TB (Caregiver1, 9-month-old, girl).

For many caregivers, one of the primary motivators for agreeing to initiate their child on TPT for MDR-TB was their own experiences of getting sick from MDR-TB and then initiating treatment. For example, one caregiver explained that she would prefer for her child to be on TPT rather than getting as ill as she had and needing to take the full course of MDR-TB treatment, which involves more tablets over a longer period:

It is important because I don’t want to see such small children go through the same [as me]. Do you understand? To cough and maybe to drink more tablets (Caregiver 2, 5-year-old girl).

Maintaining their children’s health and protecting them from developing MDR-TB outweighed challenges caregivers experienced in everyday administration or seeing their child’s response to the TPT:

DW Is it difficult for you to see your child, to see that he doesn’t like it? Is it difficult for you to force [him to take the treatment]?

C3 No! It’s for his health. So, no.

C3’s partner It’s for his health (Caregiver 3, 7-month-old boy).

### Palatability of the levofloxacin formulation

One of the primary concerns and determinants of ‘acceptability’ when administering treatment in children, is the poor taste of the treatment and children’s reluctance to ingest it. Several caregivers, who reported tasting the novel levofloxacin formulation described it in a positive way saying it smelled nice and they were curious about its taste. One of the caregivers said:

The taste is alright. For me it almost tastes like, in the beginning, like aspirin and afterwards a mint flavour (Caregiver 2, 5-year-old girl).

Although caregivers spoke positively about the flavour, most thought the taste-masking ‘mint’ flavour was inappropriate for young children and suggested a sweeter flavour (like strawberry) should be used in future. However, one caregiver said children enjoyed the ‘vapour-like’ feel and aftertaste of mint.

When asked about their experience of preparing and administering the levofloxacin formulation, caregivers often compared it to the standard of care regimen received from their local clinic. One caregiver said she and her child would fight every time she used the clinic’s TPT regimen, but her child would ask to have the levofloxacin as if it were a carbonated sugary drink:

DW She doesn’t fight you?

C4 We did fight but she drank [the treatment].

DW Ok that’s with the old [regimen you got from the clinic]?

C4 Yes that’s the old treatment. But with the second one [the study levofloxacin] she would say ‘mummy I want to drink my pill’ […] she likes the taste of the second one [the levofloxacin regimen] (Caregiver 4, 4-year-old girl).

### Administration challenges

Participants described the novel levofloxacin formulation as both more palatable, and easier to prepare and administer than the novel standard of care TPT regimen. These challenges were related to the ease with which caregivers could prepare and administer treatment in ways that ensured their child ingested the full dose. For example, when the study team arrived at one caregiver’s home to interview her, she was busy preparing the standard of care TPT regimen she had received from the local clinic, which her 2-year-old girl and 6-month-old boy were taking. This caregiver’s children had already concluded their involvement in the study, where they had received the novel levofloxacin formulation, and had restarted the standard of care regimen. We asked her to explain the process while she prepared the treatment received from their local clinic. She described the poorer taste and longer preparation time needed to administer the TPT she received from the clinic as compared to the novel levofloxacin formulation.

I grind all these pills at the same time and then I put it in a bit of yoghurt and then she eats it just like that. But these pills are very bitter […] The other pills [study levofloxacin formulation], they were better. It tasted better (Caregiver 5, 6-month-old boy & 2-year-old girl).

Further, while the caregiver had received a syringe with which to measure the old TPT regimen doses she received from the clinic, she pointed out that preparing and administering an accurate dose was complicated and took a long time.

[The clinic regimen] pills are ground and then I mix it in eight mils of water, then I only pull up three mils […] then the rest must be thrown away. These pills are also harder than those other pills and have a casing on them. That also makes it difficult because they don’t dissolve (Caregiver 5, 6-month-old boy & 2-year-old girl).

The caregiver then closely watched her daughter ingest the TPT, drinking the crushed tablets from a small cup, saying:

Swallow it [pause]. She’s gonna spit it out. Swallow it like mommy showed you with those other pills. Swallow it. Swallow! Finished! (Caregiver 5, 6-month-old boy & 2-year-old girl).

The caregiver then went on to explain that her daughter often complained about how bitter the clinic’s regimen was. When she had enough money to buy yoghurt, she told us, she would mix the clinic regimen’s ground-up pills with yoghurt to help mask the taste. Although easier for her daughter to ingest, she told us that this approach was unsustainably expensive. After she finished administering her daughter’s regimen, she then pulled her infant son onto her lap, holding his arms at his sides while he writhed and cried, and inserted a syringe of the clinic’s regimen into his mouth. Her son gurgled some of the treatment up as she carefully administered the treatment with the syringe. Together, administration for both children took well over half an hour.

Other caregivers also described the preparation and administration of the levofloxacin TPT formulation as ‘easier’ than the local clinics’ regimen, saying, “it’s easy to break, it’s also easy when you put it in the water” (Caregiver 1, 9-month-old girl). Although the novel levofloxacin formulation was far easier to prepare and administer, caregivers often still had to employ deception or bribery to ensure their child ingested the treatment. One caregiver told us that her 3-year-old daughter had become suspicious of anything she offered her to eat or drink. She had already exhausted options like crushing the pills and sprinkling them in yoghurt, she explained, as her daughter now refused any yoghurt. This meant that she had to come up with new strategies for administering her daughter’s TPT:

You have to plan and strategize ways on like, ‘how am I going to do this with her today? I must do this I must do this.’ Now I take chips then I pour them into a container, all of them, and I also take a lollipop, all the things that are nice, and I put them here and I say you are not going to get them if you don’t drink your pills (Caregiver 6, 3-year-old girl).

The constant need to ‘strategize’ brought the caregiver close to tears. She found administering the TPT to be relationally and emotionally taxing, as well as practically demanding. The relational and emotional burden compounded over time for caregivers trying to ensure long-term treatment adherence in children, resulting in feelings of powerlessness and insecurity. By contrast, children’s willingness to ingest the study TPT regimen alleviated this strain on caregivers.

In addition to the relational and emotional burden on caregivers, broader financial concerns and needs often crept in and impacted the acceptability of TPT for children in resource-constrained households. The caregiver of a 5-year-old girl disclosed to us that she had previously discontinued her child’s TPT regimen (which she received from their local clinic) due to her increased appetite:

I was not happy at all with those pills because [in] November, like I told the nurses, I stopped giving her the medication because that medication made [my daughter] eat like I don’t know what! She was [eating] out of bounds man! Continuously! The whole night as well […] she constantly wanted bread (Caregiver 2, 5-year-old girl).

In this instance the increase in food consumption put the household’s financial stability at risk and led the caregiver to discontinue her daughter’s treatment. When TPT is too difficult to integrate into caregiver’s everyday lives, including because of the pragmatic challenges of preparing and administering the treatment, or if the treatment poses risks to the wellbeing of other household members, the acceptability of the treatment regimen is diminished.

### Challenges to administering TPT as caregiver with MDR-TB

For caregivers with MDR-TB, the pragmatic challenges of administering TPT to their children are amplified by the need to manage the effects of their own treatment and illness on their bodies and everyday routines. The demands of caring for both themselves and their children often collided in the moment of administration. One caregiver of a 7-month-old boy, for example, told us that she was reminded to administer her child’s TPT immediately after returning home from receiving her own treatment at the local clinic. While her return home served as a reminder to administer her child’s treatment “every day the same time”, the adverse effects related to her MDR-TB treatment–both the injections and the 21 pills she took every day–stifled her ability to administer her child’s treatment. When the demands on her own body were too great–that is, when she was too weak, tired or, as she put it, ‘confused’–she relied on her partner’s assistance to crush the child’s pills, dissolve them in water and administer the mixture with a syringe. Her son “didn’t like it at all,” the caregiver explained, meaning that her partner had to hold him down while administering the treatment. The caregiver’s partner, who was present at the time of the interview, interjected to explain that he had to help administer the treatment because “her fingers would spasm or something like that and then I help her, or she just begins to cry” (Caregiver 3’s partner, 7-month-old boy). When we asked the caregiver about the adverse effect of her treatment, she said:

it makes me very confused sometimes […]. One moment I feel like myself. The next moment I feel completely different. I’m not myself […] it looks like I’m crazy (Caregiver 3, 7-month-old boy).

Thus, the caregiver needed to manage the side-effects of her MDR-TB treatment, whilst simultaneously navigating the pragmatic challenges of integrating her son’s TPT into her everyday household routines.

At times, the tension between caring for oneself and caring for one’s children led caregivers to suspend their own treatment or care to ensure their children received more effective care. This sacrificial caring was often linked to the financial and social costs of care. A mother of triplets, for example, recounted to us how it took her almost six months to seek care at a clinic and receive her MDR-TB diagnosis because of her primary desire to care for her children:

You always put [your children] first. You don’t even want to give R400 for the doctor for yourself […]. So I would say for six months I had the cough […] and every month I would say ‘no I’m not going to take this [money] I’m rather going to buy milk,’ […] cause you must [use] whatever money you have, you must put away for milk and nappies and you don’t want to always ask people to help you because now you have triplets (Caregiver 7, 2-year-old girls).

As she explained, her desire to be a good mother required her to put her children’s needs ahead of her own. She also stressed the importance of being seen to be taking responsibility for her three children by not asking for too much social support. Presenting oneself as a good mother or caregiver was made more complicated for the caregivers with MDR-TB in our study sample because they were also seen to be the reason for their children needing to start TPT.

Furthermore, these caregivers’ onerous experiences of their own treatment threatened to overwhelm them and cripple their ability to ensure optimal adherence to TPT in their children. We asked the caregiver of a 3-year-old girl if she thought she had the capacity to administer the full 24 weeks of treatment when restarting the ‘standard of care regimen’. “No, I don’t” she said “because I am also not well myself.” While she understood that “we are dependent on these pills now”, she also explained that her own experience of pill-taking helped her understand her child’s experience, “I understand what she’s going through because I myself don’t like pills.” (Caregiver6, 3-year-old girl).

Later in the discussion, this caregiver confessed that she lacked her husband’s support when it came to her daughter’s TPT. In fact, her husband was often hostile towards her for causing their daughter distress during TPT administration. When asked if her husband ever assisted with administering the TPT, she explained:

Oh, her dad is always at work. And other times if he is here, he will say ‘leave her alone,’ because he doesn’t want her to cry. […] I’m the only one who is in charge of the pills. I doubt he even knows. Have you seen when someone knows if you were not there, or if you are ill, how it’s done? He isn’t even interested in that because her pills just sit there. Even mine [treatment], I don’t think he has an idea. He asked me ‘how many [pills] are there, then he exclaimed ‘oh my! Twenty-something!’ I’m just saying that I don’t know how men don’t pay attention (Caregiver 6, 3-year-old girl).

The caregiver’s struggle to administer her daughter’s TPT involved an ongoing mental battle of prioritising both hers and her daughter’s treatment adherence in the face of adversity. This adversity went beyond the practical aspects of strategizing different ways of preparing and administering her daughter’s TPT. While struggling with her own treatment and having to physically fight with her daughter about the novel TPT, she also had to struggle against her husband’s apparent indifference to both her own and her daughter’s treatment. Caregivers on treatment for MDR-TB who did not receive support from their immediate family members or other social networks struggled to adhere to their own treatment regimens, greatly impacting their present and future capacity to care for their children.

### Caregivers’ experiences of stigma and its impact on their ability to ensure their child adheres to TPT

Withdrawal of social support by family or friends due to the stigma surrounding an MDR-TB diagnosis and blame for exposing household contacts to MDR-TB intersected with participants’ own feelings of guilt and shame. The caregivers with MDR-TB experienced all three domains of stigma–enacted, anticipated, and internalised–about their own diagnosis, and frequently their child’s diagnosis reinforced that experience as either a further reminder or an additional reason for being blamed. Caregivers spoke about how family members and others limited interactions with them following their diagnosis or blamed them for the ‘immoral behaviour’ assumed to be involved in exposing themselves (and their children) to MDR-TB.

#### Caregivers’ experiences of enacted stigma

One caregiver reflected on the tension between her sense of self as a moral person and other people’s perception of her MDR-TB diagnosis: “you’re trying your best to live right […] and then you just get [MDR-TB] just by being on a train or in a taxi” (Caregiver 7, 2-year-old triplets). She recounted how her partner blamed her for her MDR-TB diagnosis, “one night we argued and then he just said ‘Yes, you brought TB into this house.’ You see, he thought I was smoking with someone or whatever” (Caregiver 7, 2-year-old triplets). The caregiver described how she had to fight against prejudicial discourse that cast her TB diagnosis as shameful and raised questions about her capacity as a mother, and her fidelity to her spouse. After trying to explain the route of transmission of MDR-TB and that ‘anyone can get it,’ she also sought additional support from a social worker to try to alleviate the speculation of misconduct on her part.

For another caregiver the sense of shame and experience of stigma subsequent to her MDR-TB diagnosis was coupled with experiences of enacted stigma. The caregiver described how her sister would put a seat between them when attending the cinema, and her family forced her to use a specific mug in the house and insisted that her toothbrush be stored apart from other family member’s toothbrushes. The caregivers’ experience at home appeared to heighten her fear of possible stigmatisation outside the household.

#### Caregivers’ experiences of anticipated stigma

At the same time as managing her angst over being stigmatised at home, the caregiver feared further stigmatisation from others in the community. She admitted to crying after being diagnosed with MDR-TB, and to feeling ashamed of her diagnosis:

I still feel ashamed. I don’t want to go out. And I don’t really like the fact that you’re wearing [those] clothes either. That uniform [DTTC staff wear when collecting data] with this TB sign. I don’t like it. I don’t want people to know. I basically just hide. I don’t want family to know. I just want to be on my own (Caregiver 8, 2-year-old girl).

The caregiver also spoke about having to manage the loss of her identity as both financially independent and as a mother. After being diagnosed with MDR-TB, she had to leave her job as the manager of a restaurant. Before her diagnosis, she explained, she was able to look after her daughter and even give her ‘what she never got’ as a child. But after her diagnosis, she became financially reliant on her parents again. The loss of financial independence was coupled with a feeling of loss of control over her life. Her life, she explained, had become focused solely on managing her MDR-TB.

Her feelings of dependence were amplified by the demands of administering her daughter’s TPT treatment. When asked about how she administered TPT to her daughter, the caregiver described how her relationship with her own treatment had alienated her from her daughter, and that she had relinquished responsibility for administering her child’s TPT to her mother:

[I don’t]; my mother gives her tablets, because with so many tablets I need to drink I want to stay away from tablets […]. After my mother gives [my daughter her TPT] I can still smell the tablets on her. That’s what has pushed me a little away from my daughter. I can smell the medication, and I hate the smell of it. I must already take twenty odd tablets. So, I let my mother give her the tablets (Caregiver8, 2-year-old girl).

The caregiver’s adverse reaction to her own treatment impeded her ability to administer her daughter’s TPT. However, as with other caregivers interviewed, she later remarked that her daughter’s health and wellbeing as well as her own desire to regain her role as mother motivated her to stay on treatment despite the difficulties she faced.

#### Caregivers’ experiences of internalised stigma

The struggle to retain an identity as a good mother in a household filled with accusation and stigmatisation could be a constant challenge. Furthermore, it appeared that mothers battled with the effects of isolation and depression, commonly brought on by a TB diagnosis [38]. Caregivers’ narratives were often laden with shame at being unable to properly care for their children on various levels, including financial, emotional, and practical. Caregivers expressed managing their own disease, navigating interpersonal conflict and emotional distress whilst simultaneously trying to care for their children was a great challenge.

However, when caregivers were able to overcome the shame and stigma associated with MDR-TB, they described how adhering to their own treatment and being cured of MDR-TB was the best way to care for their children in the long-term. One caregiver recalled how she had almost given up on life, succumbing to the prejudice she had seen someone else with MDR-TB experience, when she was first diagnosed with MDR-TB two years prior to our interview:

I know where I got it. I used to live at one of my friend’s house and with the people at the back. She didn’t take her medication and so she started to give [TB] to everyone! And I was the closest to her. Her mother didn’t want to wash her. I washed her. I dressed her. I wasn’t scared of her. So, I was in their house most of the time, that’s why I picked it up quickly (Caregiver 9, 6-month-old boy).

After witnessing how her friend had been abandoned by her family, the caregiver stopped taking her own treatment, saying “I could just die”. The participants’ vicarious experience of stigmatisation through witnessing her friend’s abandonment, and probable subsequent self-stigmatisation and resignation to dying of MDR-TB was, however, recast when she found out she was pregnant. She decided she would need to care for herself to care for her son:

Now my child has to go through all this stuff because of my stupidness! Sometimes I get so frustrated, [especially] when I have to go to the hospital! […] Joh! I stressed a lot. Then I just tell myself ‘okay fine, that time [my son] wasn’t here’ […] I felt alone […] now Jakes is here, so I have to go through this [treatment]. […] It’s for my safety and for his safety (Caregiver 9, 6-month-old boy).

Although the caregiver sorely regretted her decision to discontinue her previous treatment regimen, she also recognised that at the time, her son “wasn’t here,” and “he gives me hope every day.” Like other caregivers’ narratives, the caregiver derived motivation to care for herself from her desire to better care for her son. Additionally, she had begun to receive social support from people in her local church who knew about her disease, which gave her “more courage”.

In the narratives of many caregivers their MDR-TB diagnosis initiated a cascade of negative experiences, which cumulatively affected their ability to administer TPT to their children. Caregivers with MDR-TB suffered from their own physical ailments and struggled to manage a complex treatment regimen. They also experienced blame and shame because of the stigma surrounding their diagnosis. Being blamed for their diagnosis and experiencing shame limited caregivers’ ability to activate social support. A lack of social support increased their sense of isolation and impacted their overall capacity to ensure optimal care for themselves and their children. This cascade of negative experiences could be interrupted if caregivers received sufficient social support to manage their own diagnosis. For caregivers who received greater social support they recounted feeling capable of ensuring their own and their child’s treatment adherence.

## Discussion

Overall, we found the novel levofloxacin formulation for the prevention of MDR-TB was acceptable amongst caregivers in our study sample, conventionally defined as the user’s ability to use medicine as directed [15]. Our findings contextualise and build on the quantitative findings of the lead-in acceptability study of this levofloxacin formulation, which also revealed high levels of acceptability [17]. Caregivers reported that the novel, child-friendly levofloxacin regimen was easy to prepare and administer due to its soluble formulation and overall palatability, especially when compared with the arduous preparation and administration processes required for the bitter TPT regimens they had received from their local clinics. Our findings illustrate how factors related to treatment formulation and palatability continue to play an important role in determining the acceptability and adherence to TPT. Furthermore, we found that caregivers were motivated to use the TPT as directed, despite administering it to otherwise well children.

Other studies on the experiences of caregivers administering TPT in children have, however, presented a mixed picture. In 2010, the South African government made a commitment to integrate TPT into the existing TB and HIV care programmes for children and adults. However implementation and uptake of TPT for children has remained suboptimal with about a third of children with TB going undiagnosed or unreported in South Africa [39,40]. The reasons for the slow integration of TPT into the health system include health systems barriers like limited implementation of contract tracing, poor acceptability of TPT regimens, inconsistent knowledge about TPT guidelines, and misconceptions among health workers and patients about the risks of developing drug resistance [41–45]. Caregivers in our study said they were motivated to administer TPT because they did not want the child to experience MDR-TB disease or treatment as they (the caregivers) had. Some understood TPT to protect their child from MDR-TB, while others understood TPT to build their child’s immune system and thereby ensured their child did not develop MDR-TB. This may be an important consideration when health workers communicate how TPT works to caregivers.

Studies from other low- and middle-income countries have found health education among health workers and caregivers, stigmatisation, concerns about the possibility of developing TB resistance, and uncertainty around the logic of administering treatment to well children, to impede TPT uptake and adherence in children [13,46,47]. Although caregivers in our study found it easier to prepare and administer the novel levofloxacin formulation compared to the standard of care treatment, it was often still necessary to overcome children’s ongoing resistance to treatment ingestion. Caregivers bribed, threatened, tricked, and praised children to encourage them to ingest treatment. This finding is well-established in other literature on treatment administration in children [48,49].

Despite the broad nature of the definition presented above, measures of acceptability have predominantly revolved around the palatability of drug formulations and the ease of treatment administration [35]. Our findings corroborate recent research among caregivers in Africa which suggests that the use and uptake of TPT, as well as other long-term medical conditions in children, is complicated by the psychosocial circumstances into which children’s treatment is introduced [50,51]. Other research has also illustrated how the everyday practices of caring for children is further complicated when caregivers themselves were sick [25,52]. The caregivers in our study with MDR-TB experienced additional challenges in the form of side effects, and financial and social costs related to their own treatment regimens. These caregivers had to manage the tensions between caring for themselves and their child(ren), which included relying on others to help administer their child’s TPT while they managed the adverse effects of their own treatment.

The challenges to administering TPT reported by caregivers in our study were compounded by their concurrent experience of MDR-TB disease and experiences of stigma. People with (MDR-) TB experience their diagnosis as devaluing and as a mark upon their bodies, which may negatively impact patients’ willingness to access and engage with healthcare services [53,54]. According to Naidu and colleagues [55], people with MDR-TB experienced higher levels of stigma than people with DS-TB and are at increased risk of developing depression. However, other research suggests MDR-TB stigma-related depression may be due more to underlying socio-economic needs than the MDR-TB diagnosis [38]. Caregivers with MDR-TB in our study expressed fearing anticipated stigma from others due to their diagnosis, at some point during their treatment journey, and some had internalized stigma, sanctioning pejorative feelings, prejudice or discrimination expressed by others towards people with MDR-TB [56,57]. Research among caregivers of children on antituberculosis treatment and antiretroviral treatment has explored how mothers bear the bulk of responsibility for care and often feel guilt and shame at putting their children in harm’s way [26,58]. Some research has also shown that older children may similarly internalise stigma following an MDR-TB diagnosis [59].

The mechanisms underlying stigmatisation within households in which one or more people are on preventive therapy or treatment for MDR-TB is still unclear. Yet, our findings suggest that caregivers’ ability to adhere to their own treatment and overcome the guilt, shame and stigma related to their MDR-TB diagnosis, impacted their ability to ensure their child(ren)’s optimal adherence to MDR-TPT. Qualitative research suggests that people on long-term treatment, like those with MDR-TB, weigh their psychosocial and socio-economic needs and circumstances alongside their medical or health-related needs when deciding whether to adhere treatment regimens [60]. There is some evidence to suggest that people with MDR-TB receiving appropriate socio-economic and psychological support could have significantly better health outcomes than those without support [61,62].

These are the first qualitative data looking at TPT for children exposed to MDR-TB. As such the findings have value for research on the acceptability of TPT among caregivers and their children. One of the key strengths of our study design was having several interactions with each caregiver over time, allowing us to build rapport and engagement. Further, we sampled purposively for diversity to facilitate transferability–although extrapolation to other contexts should always consider this limitation. Our sample population was drawn from a clinical trial setting where participants receive more personalised care than is possible in the public health system. Conversely, the children in this study had already been on a difficult treatment regimen. It is possible that treatment naïve children may have responded differently. Furthermore, caregivers in this sample were more likely to receive detailed explanations to any queries they may have had related to TPT and/or TB transmission and infection. This may explain why none of the caregivers in our study were less sceptical of administering TPT to their well children. Also, all but one of the caregivers in our study were mothers. Finally, although challenging in children under 5 years, the study would have benefitted from engaging with children directly alongside their caregivers about their experiences of both caring and being cared for, to further illuminate challenges particular to young people [63]. This is an opportunity for future research, particularly as there are now several TPT studies currently enrolling older children, and as guidelines are increasingly advocating for the provision of TPT to older children, adolescents, and adults [64].

We illustrated how caregivers’ everyday choices of when and how to use treatment are influenced by psychosocial circumstances as much as they are by the treatment itself. As a counterpoint, we have highlighted the everyday jeopardies of using treatment in resource constrained settings. We have argued that these complexities are even more challenging for caregivers with MDR-TB to navigate. Caregivers’ MDR-TB diagnosis initiated intra- and interpersonal tensions they must deftly navigate to ensure they can rely on others for psychosocial and financial support throughout their own and their child’s treatment period(s). Thus, the paper introduces an opportunity for new research into the impact other people’s diagnosis and treatment within the same household may have on children’s treatment overall acceptability—including treatment uptake and adherence.

Additionally, we contribute to existing research on the acceptability of TPT and TB treatment in children, by illustrating the impact contextual exigencies have on the overall acceptability of treatment in children. It is imperative that health system policies begin to acknowledge how caregivers’ household circumstances circumscribe the possibilities for children’s overall health and wellbeing. These factors include the dynamics of treatment preparation and administration in the home and the emotional, relational, and financial burden of care on the household into which treatment is introduced.

We urge drug developers and policy makers to continue to prioritise the creation and expedient roll-out of child-friendly antituberculosis treatment, that ameliorate the burden on caregivers and their households administering treatment to children. Drawing on the findings of previous research and our own study insights, we suggest that caregivers with MDR-TB should be offered psychosocial, economic and health systems support equal to their needs, to support adherence to their own treatment and their child’s TPT. This will likely require close interaction between community-based health workers and people (including caregivers) with MDR-TB. These complexities of care are probably not unique to MDR-TB, nor to the South African context, but they are probably most profoundly felt in places of resource constraint where opportunities to choose different formulations, health services and manage potential stigmatisation, are limited.

## Supporting information

S1 File This is the semi-structured discussion guide. (PDF)
