# Supplementary material for: Practical and psychosocial challenges faced by caregivers influence the acceptability of multidrug-resistant tuberculosis preventive therapy for young children
Source: PLoS One. 2022 Jul 14;17(7):e0268560. doi: 10.1371/journal.pone.0268560 (PMC9282439; doi:10.1371/journal.pone.0268560)
Supplement: S1 File — (PDF) [file pone.0268560.s001.pdf]

Date completed:

Time completed:

Unique page code:

Name of person completing form:

**(PHASE I ) Discussion Guide**

**(Parent/Guardian/Carer, Child and Nurse)**

*Purpose: The TB-CHAMP trial compares a new dispersible formulation of Levofloxicin anti-tuberculosis regimen with a placebo. The regimens are delivered in a new dispersible child-friendly formulation of levofoxicin (LFX), offering practical advantages over adult formulations, particularly for children. This study aims to understand the experiences and perceptions of nurses, parents and children making use of the new child-friendly LFX formulation. The interview guide is to facilitate discussion about key topic areas, but should be used flexibly with each participant over the course of several interactions with them. It is to be used alongside the ARF and RIDD.*

***Why are we doing this:*** *To describe the experiences of a new formulation of dispersible Levofloxicin (LFX) in a well characterised cohort of children exposed to MDR-TB, their caregivers and nurses.*

*Objectives*

- *Understanding of LFX dosing relative to pre-specified weight bands in children*
- *Understanding of dosing relative to the dispersability of the LFX*
- *Experiences of palatability and acceptability amongst children*
- *Experiences of administration in real-life (including household and clinic) contexts including co-administration of other drugs (including ARVs) and food*
- *Examine practical challenges to administering the dispersed formulation*

*Form of data recording: (1) Audio-recording of all talk from "Preamble" to "Closing". (2) Notes of key points per topic area handwritten by the facilitator into a printed copy of this document. (3) Still photographs of relevant images.*

*Expected time needed per use: 30-45 mins*

Date activity conducted: ____________________________

Place conducted: _______________________________

Time period: _______________________________

Unique page code:

*Preamble* (**to be read by facilitator**): Today is the (**insert date [day xx^th^ Xxx xxxx]**) and it is (**insert time XX:XX**). This is a discussion with (**insert participant**) who forms part of the LFX TB-CHAMP study. Thank you for your time. These discussions are part of one of the aims of the study which is to provide detailed descriptions on experiences and perceptions of making use of the new dispersible LFX formulation instead of the adult regimens. For this interview, we would like to find out more about your perceptions and experiences. May I remind you that we are audio recording this discussion and ask that you speak loud and clear? As the facilitator I will also be taking some notes. Do you have any questions before we begin?

1. *Topic area 1 – Tell us about your community*

**Please tell us a little about where you live.**

1.1. **Can you tell us about your community and what it is like living here?** What kinds of social services are available in your community (such as healthcare or security/policing/punishment for crimes? What kinds of social services are not available in your community? How many schools are there? Is there a library? How many clinics are there? What social activities happen in this community? Describe the people who live here?

1.2. **Where do you access health services?** When/why/how often do you go to the clinic? Where else do you go when you need care? Do you use alternatives before or alongside clinic health services? (e.g. self-medicating at the pharmacy, or visiting an alternative healer?)

1.3. **Can you tell me a little about the spaces and places you go in the community?** When/why/how often? Why do you decide to go to some places and not others? Why do you like to go to X place instead of Y place?

1.4. **How do you get to the places you go?** Public transport? Own car? Are there places that are difficult to access? How does transport influence where you go/how often?

2. *Topic area 2 – Tell us about your household*

**Please tell us a little about yourself, your house and your family.**

2.1. **Can you tell us more about your household?** Who lives with you? Do any of your family members also live in this community? Where was your child born?

2.2. **Are there times in the year when your household structure is different?** For example, during school holidays/in December? What are the ways that your household structure changes in these times?

2.3. **What changes have taken place in your family over time?** Has there been any losses in your family? Have any of your family members moved away?

2.4. **Who takes care of the home?** Who is responsible for it? Who cooks? Who makes the grocery list? Who goes out to buy the groceries? Who does the dishes? Who takes care of the children?

2.5. **How are health-related events part of your family's life?** How has health and illness shaped your life in this place? Has being sick ever limited your movement around or in and out of the community?

2.6. **How did you and your family deal with your own, or person living with TB in your household's diagnosis?** How was the family/household told that they have TB? Did anything in your house change after people found out?

3. *Topic area 3 – Understandings of TB*
**Please tell us a little about TB, how does one contract TB, what symptoms does TB have, and how you treat TB.**

3.1. **When I say Tuberculosis (TB), what is the first thing that comes to mind?** When did you first learn about TB?

3.2. **When did you first visit the clinic about this TB?** How did you feel about your child undergoing the TB test? Please tell us about all the tests that were performed on your child. Was there anything about these tests that they did not like or did not understand? Had you/your child been feeling ill for a while before visiting the clinic? When did you notice that you/your child was feeling unwell? When did the nurse tell you that it was TB?

3.3. **What was your reaction when you heard that it was TB?** Do you know anyone else who has/had TB? What do you think the differences are in the treatment your child is receiving compared to them? Which form of medication do you prefer?

3.4. **What did you think about the treatment after the nurse explained how you should be taking it?** Has it been as easy/difficult as the nurse said it would be? What have some of the most difficult challenges been? When during the day will you be taking the tablet?

4. *Topic area 4 – Palatability and acceptability of dispersible LFX*
**Please tell us about the TB treatment that you are receiving.**

4.1. **Please tell us more about your child's TB treatment journey?** What are some of the difficulties and challenges you and the child experienced during the treatment process? Are there any problems with the medicine (side effects)? If yes, what are they and what do you do? Do you have any questions regarding these side-effects, if yes, what questions do you have about your child's treatment?

4.2. **How do you think your child feels about taking the medication?** Do you have to convince your child to take the treatment? (For example, do you have to bribe them?) Or use force? Or distract them? Or reward them?

***If/when speaking to the child* *These questions can also be posed to the parents of younger children****

4.3. **Can you tell us the story about how you found out you had TB?** How did you feel before being told that you have TB? Was there any part of your body that was sore? What did the nurses/doctors say to you when they told you that you have TB? How did you feel; were you scared?

4.4. **After the nurse/doctor explained that the person in your household has MDR-TB HOW did they explain the treatment they are giving your children to you?** How did they explain the treatment Did they tell you a story about how the medication tries to prevent you from getting TB? Was this helpful?

4.5. **How do you feel when you have to take the treatment, or when you have to give the treatment to your child?** What time of the day do you take/give the treatment? How do you remind yourself to take/administer the treatment? Does anyone help you? Who/when/why?

4.6. **What does it taste like?** Does it matter if your child has already eaten something before/after taking the tablet? What are some of the worst tasting things that you can't stand to eat/drink, and how does this tablet compare?

4.7. **Do you think it would be better to swallow the pill whole or is better to dissolve it in water?** How much water do you use to dissolve it in? Do you think there are other ways to take the medication?

4.8. **What do you use to measure or break-off the tablet?** Do you cut the tablet with a knife, or do you break it with your hand, or how? Can you demonstrate it for me?

4.9. **If you were going to tell another child/mother about this tablet, how would you describe it?** What ideas do you have to explain what it tastes like?
